# Genetic Variant of C-5434T *REN* Enhancer on Serum Renin Levels and Binding Pattern of Signal Transducers and Activators Transcription 3

**DOI:** 10.1155/2015/486961

**Published:** 2015-05-21

**Authors:** Imama Maslahah, Mohammad Saifur Rohman, Nashi Widodo, Agustina Tri Endharti, Didik Utomo

**Affiliations:** ^1^Biomedical Sciences, Faculty of Medicine, University of Brawijaya, Malang 65145, Indonesia; ^2^Department of Cardiology and Vascular Medicine, Faculty of Medicine, Brawijaya University, Saiful Anwar General Hospital, Malang 65145, Indonesia; ^3^Biology Department, Faculty of Mathematics and Sciences, University of Brawijaya, Malang 65145, Indonesia; ^4^Parasitology Department, Faculty of Medicine, University of Brawijaya, Malang 65145, Indonesia

## Abstract

The human renin gene has been widely known to be involved in essential hypertension (EH) pathogenesis. Genetic variant C-5434T of *REN* enhancer contributed to renin gene transcription and serum renin regulation. However, the mechanism associated with the transcription level changes remains unknown, and only a few reports exist that discussed serum renin levels on C-5434T of *REN*. Thus, this study aims to investigate the relationship between genetic variant C-5434T of *REN* enhancer and serum renin levels in Indonesian hypertensive patients. SNP of C-5434T was genotyped in 56 hypertensive patients by using RFLP. The data showed that serum renin is slightly higher in hypertensive patients with the TT genotype (39 ± 10.3) than patients with the CC genotype (33 ± 10.6) but the difference was not statistically significant (*p* = 0.35). Here, we also present a docking approach for predicting interaction between genetic variant -5434C/T and STAT3 (Signal Transducers and Activators Transcription 3), the predicted transcription factor that regulates renin gene enhancer. The results showed that STAT3-DNA allele T more favorably binds to DNA than STAT3-DNA allele C. These data suggest that the presence of genetic variant C-5434T has changed the binding pattern of STAT3 to *REN* enhancer. This is likely to influence STAT3 activity to stimulate the expression of renin gene in producing renin.

## 1. Introduction

Renin-angiotensin-aldosterone system (RAAS) plays a pivotal role in blood pressure regulation. Renin, the key enzyme of the renin-angiotensin-aldosterone cascade, plays a crucial role in the regulation of blood pressure, and* REN* may be a candidate gene for hypertension [1, 2]. Much progress has been made in elucidating the molecular mechanisms involved in* REN* expression [3]. Previous study showed that the presence of genetic variants within a distal enhancer region has been reported to increase in vitro* REN* transcription [4].

A SNP C-5434T is one of the variants to be found within distal enhancer region (nucleotides -5777 to -5312) [4]. We hypothesized that this variant can affect the basal transcriptional activity in generating renin. However, in Indonesian population, there is no report that investigates the relationship between genetic variant C-5434T of* REN* enhancer and serum renin levels.

Transcription factors bound to the* REN* enhancer elements can have the effect of increasing the* REN* transcription, thus indicating that the transcription factor has a crucial role in the regulation of gene transcription [5]. One of the transcription factors that regulate transcription activity in* REN* is STAT3 (*Signal Transducers and Activators Transcription* 3) [6].* REN* enhancer has a motif DNA sequence for STAT3 binding in the STAT-binding element (SBE), 5′-TT(N_5_)AA-3′. STAT3 is thought to be the major transcription factor in genetic variant -5434* REN*.

Therefore, this study was designed to investigate the possible role of the genetic variant C-5434T in regulating the expression of* REN* and confirmed by serum renin levels in hypertensive patients in Indonesia.

## 2. Materials and Methods

### 2.1. Subjects

Fifty six patients with hypertension at the Outpatient Clinic of Dr. Saiful Anwar General Hospital, Malang, Indonesia, were enrolled in this study. Patients with any form of secondary hypertension, overt renal insufficiency (serum creatinine >1.5 mg/dL), pregnancy, and estrogen and corticosteroid therapy were excluded. The age, gender, BMI (kg/m^2^), SBP, DBP, smoking status, and normal laboratory values for physiological homeostasis were required.

### 2.2. Detection of Polymorphism

Genomic DNA samples were identified by means of a PCR, followed by a RFLP. The PCR product was amplified to 376 bp using 5′CGTAGTGCCATTTTTAGGAAC3′ and 5′TTTCTACTTACCAAATGGCGTC3′. The RFLP products were incubated at 65°C for 5 hours. The presence of renin gene polymorphism resulted in a loss of the MaeII restriction site (5′-ACGT-3′). The digested fragments were separated on a 1.5% agarose gel.

### 2.3. Renin Levels

Serum samples of all patients were screened using indirect ELISA with the following renin (A-1): sc-137252 as first antibodies and rabbit anti-human IgG-HRP (sc-2769) as secondary antibodies.

### 2.4. Starting Structure of DNA and STAT3

A double-stranded 18 bp DNA was built using 3D-DART provided by HADDOCK (http://haddock.science.uu.nl/services/3DDART/) [7]. We generated 3D structural models of DNA from sequences of* REN* enhancer (5′-AGTTTTACTAGAACGTAG-3′) for allele C and (5′-AGTTTTACTAGAATGTAG-3′) for allele T. The homology model of STAT3 human was constructed using USF Chimera. The structure from protein structure database under the PDB ID number 1BG1 was chosen as a template for modeling.

### 2.5. Docking Procedure

STAT3 and DNA were docked using the docking program HADDOCK (http://haddock.science.uu.nl/services/HADDOCK/) [7, 8]. HADDOCK was run using its default but with additional information about active site of DNA (T5, T6, A7, C8, T9, 10A, G11, A12, A13, and C14/T14) and a list of amino acids that might be involved in interactions with the DNA (M331, H332, K340, T341, V343, Q344, R382, E415, R417, R423, I431, V432, S465, N466, and I467). The best 40 complex structures were selected on the basis of HADDOCK score. Then, the complex of docking result was analyzed using NUCPLOT to know the amino acids and nucleotides responsible for the interaction.

### 2.6. Analysis

Baseline characteristics and serum renin levels finding between 3 groups (CC, CT, and TT) were compared by one-way ANOVA test for parametric and Chi-square test for nonparametric analysis. For all tests, a *p* value ≤0.05 was considered statistically significant. Statistical analysis was performed with SPSS 16.0. For DNA-protein docking we analyzed descriptively binding pattern and protein-DNA contacts.

### 2.7. Ethics

The study was approved by the local committee on medical research ethics. Written informed consent was obtained from all study participants.

## 3. Results

### 3.1. Baseline Characteristics

The baseline characteristics of hypertensive patients between 3 groups (CC, CT, and TT) are summarized in Table 1. Statistical analysis showed that there was no significant difference at each baseline.

### 3.2. Analysis of Genetic Variation

We amplified a 376 bp region (-5547 to -5172) using PCR followed by a RFLP. Only one SNP was identified, at position -5434 (C→T). The genotype frequencies in this population were 28.6% for the CC genotype, 55.4% for the CT genotype, and 16% for the TT genotype. The allele frequencies were 56% for C allele and 44% for T allele.

### 3.3. Renin Serum Level and Genetic Variant C-5434T* REN*

Fifty six patients were divided into 3 groups according to genotyping result (CC, CT and TT). Then, renin levels of each patient was measured using indirect elisa method. Statistical analysis (Table 2) showed that serum renin levels were higher in patients with TT genotype (39 ± 16.46) than patients with CC genotype (33 ± 10.63) and CT genotype (36 ± 7.77) but the difference was not statistically significant (*p* = 0.35).

### 3.4. STAT3 Protein-DNA Docking

**Figure 1:**
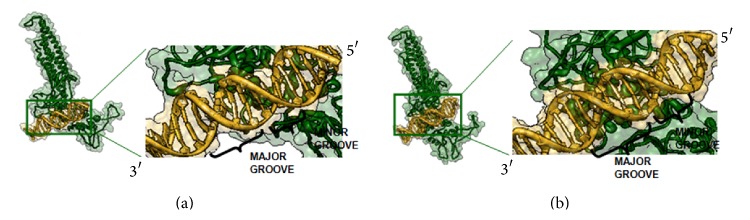
The differences of binding pattern in STAT3-DNA allele C (a) and STAT3-DNA allele T (b) interactions.* STAT3 protein (dark green) with DNA (gold)*.

A monomeric structure of STAT3 was used in this study. This structure consists of coiled-coil domain, DNA binding domain, and SH2 domain. In order to directly study the interactions between STAT3 and DNA, the monomeric structure of this protein was docked onto its DNA consensus sequence. The docking results showed that DNA binding domain of STAT3 was directly in contact with the major groove of DNA allele T. In contrast, there were changes in the conformation of the binding pattern; DNA binding domain only focused on the minor groove of DNA allele C (Figure 1). Furthermore, we analyzed in more detail the differences of complex docking using NUCPLOT program. The specific amino acid-nucleotide contacts were analyzed (Table 3). Most of the amino acids in complex STAT3 and DNA allele C bond were unfavorable contacts, but complex STAT3 and DNA allele T bond were favorable contacts in Arg423 residue with guanine 29. Overall analysis of the docking results showed that STAT3 more preferentially binds to -5434T variant than to -5434C variant.

## 4. Discussion

Renin-angiotensin system (RAS) plays a pivotal role in the maintenance of blood pressure [9]. Molecular variants of the renin gene thought to be a genetic risk factor may be involved in the etiology of hypertension [10]. In this study, we found the presence of SNP C-5434T genetic variant in our population. Then, we investigated the relationship between genetic variant C-5434T of* REN* enhancer and serum renin levels in Indonesian hypertensive patients. The data showed that serum renin levels were higher in patients with TT than patients with CC and CT genotype but the difference was not statistically significant.

Contributions of renin in hypertension are associated with the presence of genetic variation in this gene. The existence of a wide range of genetic variations affects the* REN* transcriptional activity in producing renin [11]. Previous studies which identified three variants of the renin gene, namely, SNP T_17int4G, VNTR in intron 7, and missense mutation in exon 9 (G105A), in the Japanese population showed that the missense mutation in exon 9 affects the enzymatic function of renin [10]. On the other hand they suggested that -5312, -5434, and A* Bgl*I G polymorphisms of renin gene might play an important role in the occurrence of arterial hypertension (AH). But a single analysis of the C-5434T found no significant association with the incidence of AH [12].

Here, we also present a docking approach for predicting interaction between genetic variant -5434C/T and STAT3. Based on binding pattern (see Figure 1), we found that STAT3 more preferentially binds to DNA in SBE sequence (in allele T).

The protein that interacts on the major groove of B-DNA, especially in binding sequence, shows a more functional group that identifies base pairs. The major groove of DNA is rich in chemical information compared to minor groove and is important for recognition by nucleotide sequence specific binding protein [13, 14]. The difference in the STAT3 binding in allele C (focused on the minor groove) suggests that STAT3 loses contact with the center of SBE sequence, which is very likely not able to stimulate transcription of* REN*. Then we focused on contact residues analysis between STAT3 and DNA allele C/T complexes. Active residues of STAT3 are Arg417 and Arg423 [15]. In this study, we found that most of the amino acids in complex STAT3-DNA allele C bond were unfavorable contacts. In contrast, STAT3-DNA allele T bond was a favorable contact in Arg423 with G29. Interaction between arginine or lysine and guanine gives favorable contacts [16] and the presence of arginine residue mediates the nuclear translocation of STAT3 [17]. This result might explain why the renin level of* REN* in -5434T was higher compared to -5434C.

However, in our study, the number of subjects examined was too small. Moreover, an adequate number of samples are needed in further study to analyze the statistical significance of the association between genotype and phenotype. Besides that, it is interesting to analyze transcription level of STAT3 using in vitro studies to validate the in silico result.

## 5. Conclusion

*REN* is considered as one of the target genes that are directly regulated by STAT3. STAT3 more favorably binds to DNA in SBE sequence allele T than allele C. Thus, the presence of genetic variant C-5434T can change the binding pattern of STAT3 to* REN* enhancer. This is likely to influence STAT3 activity to stimulate transcriptional activity in producing renin.

## Figures and Tables

**Table 1:** Baseline characteristics of the patients.

Variable	*REN* C-5434T			*p* value^∗^
	CC (*n* = 16)	CT (*n* = 31)	TT (*n* = 9)	
Age (years)	57 ± 6.73	59 ± 8.77	58 ± 6.98	0.57
Gender (M/F)	7/9	14/17	7/2	0.19
SBP (mmHg)	138 ± 15.43	142 ± 18.87	140 ± 10.54	0.78
DBP (mmHg)	89 ± 10.62	84 ± 11.15	87 ± 6.66	0.21
Weight (kg)	65 ± 13.20	66 ± 11.24	67 ± 10.54	0.87
Height (cm)	157 ± 7.68	158 ± 8.20	161 ± 8.55	0.45
BMI (kg/m^2^)	27 ± 4.78	26 ± 3.68	26 ± 2.98	0.97
Urea (mg/dL)	29 ± 9.18	29 ± 15.93	28 ± 8.67	0.95
Creatinine (mg/dL)	1.4 ± 1.27	0.9 ± 0.22	1 ± 0.26	0.13
Blood glucose (mg/dL)	98 ± 28.32	95 ± 9.61	91 ± 9.69	0.58
Smoking	1/15	0/31	1/8	0.22
Cholesterol (mg/dL)	182 ± 59.86	185 ± 38.38	181 ± 35.87	0.96

SBP: systolic blood pressure; DBP: diastolic blood pressure; BMI: body mass index; ^∗^ *p* value ≤ 0.05: significantly different between groups.

**Table 2:** Comparison of genetic variant *REN* C-5434T and serum renin level.

Variable	*REN* C-5434T			*p* value^∗^
	CC (*n* = 16)	CT (*n* = 31)	TT (*n* = 9)	
Renin concentration (pg/mL)	33 ± 10.63	36 ± 7.77	39 ± 16.46	0.35

^∗^ *p* value ≤ 0.05: significantly different between groups.

**Table 3:** Protein-DNA contacts (hydrogen bonds) observed in STAT3-DNA allele C/allele T.

STAT3-DNA allele C			STAT3-DNA allele T		
Acceptor	Residue contact	Description	Acceptor	Residue contact	Description
T5	Gln344 NE2	*Unfavorable contact *	T4	Gln344 NE2	*Unfavorable contact *

T6	His332 NE2 Lys340 NZ	*Unfavorable contact *	T5	Gln344 N	*Unfavorable contact *

A7	Lys340 NZ	*Unfavorable contact *	T6	Lys340 NZ	*Unfavorable contact *

A13	Arg417 NH1	*Unfavorable contact *	T14	Asp427 N Ala428 N	*Unfavorable contact *

C14	Arg423 NE	*Unfavorable contact *	T27	Thr433 OG1 Gln469 NE2	*Unfavorable contact *

G15	Arg423 NH1	*Unfavorable contact *	**G29**	**Arg417 NE, NH2 **	***Favorable contact***

A28	Arg417 NH2	*Unfavorable contact *	T30	Arg417 NH2	*Unfavorable contact *

T27	Arg382 NH1 Val432 Gln469 NE2	*Unfavorable contact *			

## References

[1] Doris P. A. (2002). Hypertension genetics, single nucleotide polymorphisms, and the common disease: common variant hypothesis. *Hypertension*.

[2] van Vark L. C., Bertrand M., Akkerhuis K. M. (2012). Angiotensin-converting enzyme inhibitors reduce mortality in hypertension: a meta-analysis of randomized clinical trials of renin-angiotensin-aldosterone system inhibitors involving 158 998 patients. *European Heart Journal*.

[3] Petrovic N., Black T. A., Fabian J. R. (1996). Role of proximal promoter elements in regulation of renin gene transcription. *Journal of Biological Chemistry*.

[4] Fuchs S., Philippe J., Germain S. (2002). Functionality of two new polymorphisms in the human renin gene enhancer region. *Journal of Hypertension*.

[5] Pan L., Jones C. A., Glenn S. T., Gross K. W. (2004). Identification of a novel region in the proximal promoter of the mouse renin gene critical for expression. *The American Journal of Physiology: Renal Physiology*.

[6] Castrop H., Höcherl K., Kurtz A., Schweda F., Todorov V., Wagner C. (2010). Physiology of kidney renin. *Physiological Reviews*.

[7] de Vries S. J., van Dijk M., Bonvin A. M. J. J. (2010). The HADDOCK web server for data-driven biomolecular docking. *Nature Protocols*.

[8] Wassenaar T. A., van Dijk M., Loureiro-Ferreira N. (2012). WeNMR: structural biology on the grid. *Journal of Grid Computing*.

[9] Perazella M. A., Setaro J. F. (2003). Renin-angiotensin-aldosterone system: fundamental aspects and clinical implications in renal and cardiovascular disorders. *Journal of Nuclear Cardiology*.

[10] Hasimu B., Nakayama T., Mizutani Y. (2003). Haplotype analysis of the human renin gene and essential hypertension. *Hypertension*.

[11] Moore N., Dicker P., O'Brien J. K. (2007). Renin gene polymorphisms and haplotypes, blood pressure, and responses to renin-angiotensin system inhibition. *Hypertension*.

[12] Chikhladze N. M., Samedova K. F., Sudomoina M. A. (2008). Contribution of CYP11B2, REN and AGT genes in genetic predisposition to arterial hypertension associated with hyperaldosteronism. *Kardiologiia*.

[13] Lodish H., Berk A., Zipursky S. L. (2000). *Molecular Cell Biology*.

[14] Watson J., Baker T. A., Bell S. P., Gann A., Levine M., Losick R. (2004). Maintenance of the genome. *Molecular Biology of the Gene*.

[15] Becker S., Groner B., Müller C. W. (1998). Three-dimensional structure of the Stat3*β* homodimer bound to DNA. *Nature*.

[16] Lustig B., Jernigan R. L. (1995). Consistencies of individual DNA base-amino acid interactions in structures and sequences. *Nucleic Acids Research*.

[17] Ma J., Zhang T., Novotny-Diermayr V., Tan A. L. C., Cao X. (2003). A novel sequence in the coiled-coil domain of Stat3 essential for its nuclear translocation. *Journal of Biological Chemistry*.

